# Comparison of the Bacterial Composition and Structure in Symptomatic and Asymptomatic Endodontic Infections Associated with Root-Filled Teeth Using Pyrosequencing

**DOI:** 10.1371/journal.pone.0084960

**Published:** 2013-12-30

**Authors:** Annette Carola Anderson, Ali Al-Ahmad, Fadil Elamin, Daniel Jonas, Yousra Mirghani, Markus Schilhabel, Lamprini Karygianni, Elmar Hellwig, Ateequr Rehman

**Affiliations:** 1 Department of Operative Dentistry and Periodontology, Albert-Ludwigs-University, Freiburg, Germany; 2 Khartoum Center for Research and Medical Training, Khartoum, Sudan; 3 Institute of Environmental Medicine and Hospital Hygiene, Albert-Ludwigs-University, Freiburg, Germany; 4 Institute of Clinical Molecular Biology, Christian-Albrechts-University Kiel, Kiel, Germany; University of Toronto, Canada

## Abstract

Residual microorganisms and/or re-infections are a major cause for root canal therapy failure. Understanding of the bacterial content could improve treatment protocols. Fifty samples from 25 symptomatic and 25 asymptomatic previously root-filled teeth were collected from Sudanese patients with periradicular lesions. Amplified 16S rRNA gene (V1-V2) variable regions were subjected to pyrosequencing (FLX 454) to determine the bacterial profile. Obtained quality-controlled sequences from forty samples were classified into 741 operational taxonomic units (OTUs) at 3% dissimilarity, 525 at 5% dissimilarity and 297 at 10% dissimilarity, approximately corresponding to species-, genus- and class levels. The most abundant phyla were: *Firmicutes* (29.9%), *Proteobacteria* (26.1%), *Actinobacteria* (22.72%), *Bacteroidetes* (13.31%) and *Fusobacteria* (4.55%). Symptomatic patients had more *Firmicutes* and *Fusobacteria* than asymptomatic patients, while asymptomatic patients showed more *Proteobacteria and Actinobacteria*. Interaction of disease status and age was observed by two-way ANOSIM. Canonical correspondence analysis for age, tooth restoration and disease status showed a correlation of disease status with the composition and prevalence of different members of the microbial community. The pyrosequencing analysis revealed a distinctly higher diversity of the microbiota compared to earlier reports. The comparison of symptomatic and asymptomatic patients showed a clear association of the composition of the bacterial community with the presence and absence of symptoms in conjunction with the patients’ age.

## Introduction

Apical periodontitis, an inflammatory process around the apex of the tooth root, is mainly caused by polymicrobial infection of the root canal. The persistence of microorganisms after endodontic treatment or reinfection of the canal due to an inadequate filling or coronal restoration often leads to post-treatment continuation of the disease as secondary infection [1]. Studies investigating endodontic failures applying culture methods, PCR and PCR cloning techniques [2–10] have reported the presence of microorganisms in 35-100% of the cases with periradicular lesions. 

The microbiota of root-filled teeth with periapical lesions differs distinctly from the microbiota of primary infections, although both harbour mostly polymicrobial communities. Previous analyses of primary infections with culture and culture-independent methods revealed nine different phyla with the highest species richness for the *Firmicutes*, followed by *Bacteroidetes* and *Actinobacteria*. More gram-negative and gram-positive obligate anaerobes were found in primary infections than in previously root-filled teeth [11,12]. In contrast, previous studies of secondary infections revealed mostly gram-positive bacteria, predominantly facultative and only few obligate anaerobes. The main phyla found are *Firmicutes*, *Actinobacteria* and *Proteobacteria*, *Bacteroidetes* and *Fusobacteria* as well as *Spirochaetes* and *Synergistes* [2,11].

Results from several culture and molecular studies demonstrated significant associations between symptoms and certain bacterial species mainly in primary infections. Examples include associations between pain and the occurrence of obligate anaerobes as well as tenderness to percussion with *Peptostreptococcus*, *Porphyromonas* and *Prevotella* species [13–17].

Both persistent and secondary post-treatment infections, originate from different causes. In persistent infections, insufficient root debridement during initial endodontic treatment allows microbial species to reinstate the disease. In secondary infections the disease-causing microbial agents may not be the same as in the primary infection. The infecting microorganisms invade the root canal after initial treatment through several possible pathways such as inadequate coronal seals, caries, fractures or loss of final restorations [1,18] resulting in post-treatment apical periodontitis that persists for years. Diagnosis is usually made by routine clinical and radiographic examination or symptomatic presentation.

Knowledge of specific etiological microbial agents in post-treatment endodontic infections, helps in improving treatment strategies. Earlier studies of microbiota in root-filled teeth with periradicular lesions have relied primarily on culture methods and biochemical identification [2,3,8,19]. However, these methods led to an underestimation of the microbial diversity due to the large percentage of uncultivable oral bacteria. More recently molecular methods have been applied to analyze the endodontic flora, using species-specific PCR, Real-Time PCR and checkerboard DNA-DNA hybridization assays [5,7,10,20–24]. These methods are usually capable of detecting targeted species only, with bacterial species selected based on earlier studies using culture methods. Few studies based on open-ended 16S rDNA cloning methods have reported new taxa that have been recovered in root-filled teeth for the first time [6,25–27]. With the introduction of high-throughput sequencing techniques, e.g. pyrosequencing, less expensive and deep-coverage analysis of microbial communities is accessible and technically feasible. Tagged pyrosequencing can achieve massive parallel analysis of microbial community profiles based on small fragments of the 16S ribosomal RNA sequences [28,29], allowing for detection of abundant and low-abundant species. This technology has been used to assess the microbial communities in various environments as well as different human body sites including the oral cavity [30–32]. Some studies investigated the bacterial composition of endodontic infections but were restricted to primary infections [33–37]. Knowledge of the composition of the microbial flora responsible for persistent and secondary endodontic infections is imperative to improve our understanding of the pathogenesis and endodontic re-infection treatment. This is the first systematic pyrosequencing approach on secondary and persistent endodontic infections. It attempts to assess and evaluate the bacterial composition and structure in previously root-filled canals in relation to different disease characteristics. 

## Material and Methods

### Clinical Material

Fifty patients who had been referred to the Khartoum Centre for Research and Medical Training, (Khartoum, Sudan), for endodontic retreatment participated in this study. All of the patients gave their written informed consent to the study protocol. The study was approved by EL-Razi College for Medical Sciences ethical committee (Ref. KCRMT/Nov2011). Patients’ exclusion criteria were: 1) severe systemic disease, 2) poor tooth prognosis, 3) pregnancy or lactation, 4) participation in any other clinical study within the last 30 days. All teeth showed radiographic signs of apical periodontitis and were deemed unsuccessful. No direct exposure was evident in any of the cases between the root filling material and the oral cavity. Twenty five patients showed symptoms, i.e. pain and/or tenderness to percussion whereas twenty-five patients had only radiographic symptoms with no clinical signs. Teeth with obturation material not within 4 mm of the radiographic apex or could not be fully isolated with a rubber dam were excluded from the study. 

### Sampling Procedure

All samples were collected under strictly aseptic conditions. Samples for bacterial growth were transferred into vials containing 0.75 ml reduced transport fluid (RTF) [38] and stored at -80°C. The sampling procedure was conducted as described in earlier studies [9,10]. The tooth and surrounding tissues were cleaned with 30% hydrogen peroxide (H_2_O_2_) and swabbed with a 3% sodium hypochlorite solution (NaOCl). Endodontic access was achieved with a sterile high-speed carbide bur until the root filling was exposed. The tooth and the adjacent rubber dam were then disinfected a second time using 30% hydrogen peroxide (H_2_O_2_) and 3% sodium hypochlorite solution (NaOCl). The cavity was swabbed with a 5% sodium thiosulfate solution to inactivate the NaOCl. A sterile foam pellet was moistened in a sterile 0.9% NaCl solution and used to swab the access cavity and the tooth surface to assess the efficacy of the disinfection. If bacterial growth occurred in these quality control samples, the tooth was excluded from the study. 

Coronal gutta-percha was removed with Gates-Glidden drills. The working length was established radiographically and with the aid of an electronic apex locator (Raypex 5; VDW, Munich, Germany). The canal was enlarged from 0.5 to 2 mm from the radiographic apex with ProTaper NiTi instruments (Dentsply Maillefer, Ballaigues, Switzerland). Teeth that could not be instrumented to this length were excluded from the study. No solvent was used at any time. After introducing approximately 40 µl sterile saline solution (0.9% NaCl) into the canal with a sterile syringe, three sequential sterile paper points ISO 25, taper 04 (ROEKO, Langenau, Germany) were placed to the working length to soak up the fluid. Each paper point was kept inside the canal for 1 minute and then transferred into a sterile vial containing 0.75 ml RTF. Conventional retreatment was finished after root canal disinfection, and the root canal filled with lateral condensation.

### DNA-extraction

Total bacterial DNA was isolated from the samples in RTF. The samples were vortexed for 2 minutes and the suspensions were then transferred to a new vial. Samples were centrifuged at 16.000 g for 10 min and the supernatant discarded. The DNA was subsequently purified with QiaAMP Micro Kit (Qiagen, Hilden, Germany) according to the manufacturer’s protocol for tissue samples. Enzymatic lysis of microbial cells was performed using buffer according to the manufacturer’s recommendations with an incubation time of 2 h at 37°C. The microbial DNA was eluted in 35 µl AE buffer (Qiagen) and then stored at -20°C.

### Pyrosequencing of 16S rRNA gene

The bacterial community and structure was analysed using 16S rRNA gene hypervariable region V1 and V2. This region was amplified using fusion primer pair 27F and 338R [39]. Specific and unique 10 base long multiplex identifier (MID) was included in the reverse primer to assign the sequences to their respective samples during analysis. The PCR reaction was duplicated for each sample, consisting of 2.5 µl of 10 X PCR buffer solution, 2 µl of 2.5 mM dNTPs, 2 µl of MgCl_2_ (25 mM) solution, 1 unit Taq DNA Polymerase (Applied Biosystems, Life Technologies, Darmstadt, Germany), 0.2 µM of each primer, and 1 µl of dimethylsulfoxide (DMSO). PCR cycles with initial denaturation at 95°C for 9 min were performed followed by 30 cycles (95°C for 10 s, 55°C for 30 s, 72°C for 30 s) and a final elongation at 72°C for 7 min. Amplicons were visualized by gel electrophoresis and specific bands of about 400 bases were fetched and purified by MinElute Gel Extraction Kit (Qiagen). Quant-iT PicoGreen dsDNA Assay Kit (Invitrogen, Life Technologies, Darmstadt, Germany) were used to determine the concentration of purified amplicons. Equal amounts of PCR amplicons from each sample were pooled and sequenced (Institute of Clinical Molecular Biology, Kiel, Germany) using Roche 454 titanium chemistry.

### Pre-processing and analysis of sequences

Obtained sequences were screened and filtered for quality control using the following parameters: sequence reads with less than 150 bases, with less than 35 average quality score in the rolling window size 50, with any ambiguous base and with more than eight homopolymers were removed. High quality sequences were aligned against the SILVA database [40] and filtered to have similar portions of the 16S rRNA gene for all sequences in the alignment. Suspected chimeric sequence reads were identified by UCHIME [41] algorithm based on SILVA reference data base implemented in MOTHUR [42] and removed from further analysis. Down-stream analysis included 40 samples with at least 450 sequences per sample, 17 samples from symptomatic infections and 23 samples from asymptomatic infections. Four hundred and fifty sequences were picked randomly from these samples to normalize the read distribution and sampling effect. 

### Phylogenetic classification

Well curated and quality controlled sequences from 40 samples were classified from domain to genera level using RDP (Ribosomal Database Project) multiclassifier [43]. A cutoff value of 50% bootstrap was used as suggested for less than 250 base sequence reads. 

### Operational taxonomical-based analysis

Sequences with at least 97% and 95% similarity were clustered into species- and genera-level operational taxonomic units (OTUs), approximately corresponding to species-, genus- and class levels, using average neighbour algorithm [44]. Phylogenetic affiliation of each OTU was governed by using RDP taxonomy and template file to link our retrieved OTUs to the nearest clone sequence in the RDP database. Statistical diversity parameters i.e nonparametric Shannon, Simpson, ChaoI and Goods estimation of coverage were also calculated by MOTHUR. Phylogenetic diversity within samples was calculated from aligned file on neighbour joining tree generated by clear-cut command in MOTHUR. Sequences from all samples are available in the National Center for Biotechnology Information (NCBI) NIH Short Read Archive with the following accession number: SRP029320.

### Multivariate analysis

For multivariate analysis OTU matrices were converted to percentage and square root transformed to reduce the effect of abundant OTUs. Canonical correspondence analysis was performed to identify the correlation of environmental factors (age, filling and disease status) on observed microbial community and composition (abundance or presence or absence of OTUs). A two-way crossed analysis of similarity (ANOSIM) was used to determine if the microbial communities were significantly different between age, filling and disease status. One-way ANOSIM was used to test if microbial communities were significantly different between groups according to disease status regardless of age or filling. It calculates *R* as a statistic value that describes the level of similarity between each pair in the ANOSIM. Values close to unity indicate completely different communities in two groups while a zero value indicates complete overlap or similarity. Similarity percentages (SIMPER) analysis was performed to determine the OTUs responsible for significant differences in bacterial community composition between two groups. Multivariate analyses were performed by PAST software [45].

## Results

### Bacterial taxa in secondary endodontic infections

Multiplexed barcoded pyrosequencing of 40 specimens of previously root filled teeth with periapical lesions yielded 38,035 sequences from partial 16S rRNA gene (V1-2 region) that passed stringent quality control. The two negative controls contained 163 sequences. After normalization (450 sequences per sample) 18,163 sequences with an average length of 150 bp were used for further analysis. The characteristics of the patients that provided the specimens are described in Table 1. 

**Table 1:** Distribution of patients’ characteristics.

**Characteristics**	**n of patients (%)**
**Gender**	
Females	31 (77)
Males	9 (22)
**Age**	
≤ 30 years	17 (42)
> 30 years	23 (57)
**Adequacy of previous filling**	
Adequate	26 (65)
Inadequate	14 (35)
**Symptoms**	
Symptomatic	17 (42)
Asymptomatic	23 (57)
**total**	40 (100)

All patients’ teeth were previously root filled, showed radiographic signs for apical periodontitis and retreatment of the root canal was indicated.

**Figure 1:**
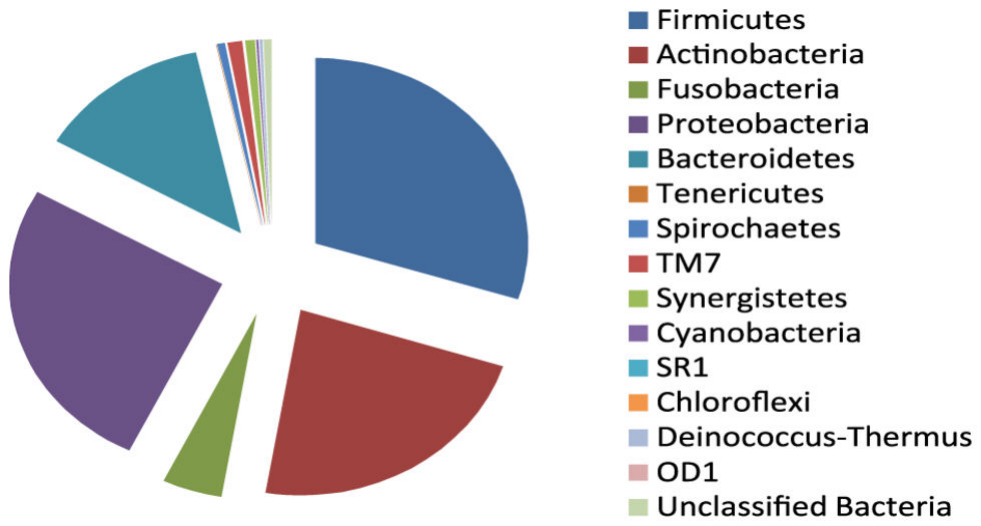
Abundance of the different bacterial phyla detected in clinical samples of root-canal treated teeth.

Overall, 14 phyla were detected in the root canal samples (Figure 1). The most abundant were *Firmicutes* (29.9%), *Proteobacteria* (26.09%), *Actinobacteria* (22.72%), *Bacteroidetes* (13.31%) and *Fusobacteria* (4.55%). Several phyla and candidate phyla were found that were not previously reported in secondary endodontic infections: *TM7*, *Deinococcus*-*Thermus*, *Cyanobacteria*, *Chloroflexi*, *SR1* and *OD1* constituted 1.5% of the sequences. About 0.6% of the sequences could not be ascribed to any phylum. 

**Figure 2:**
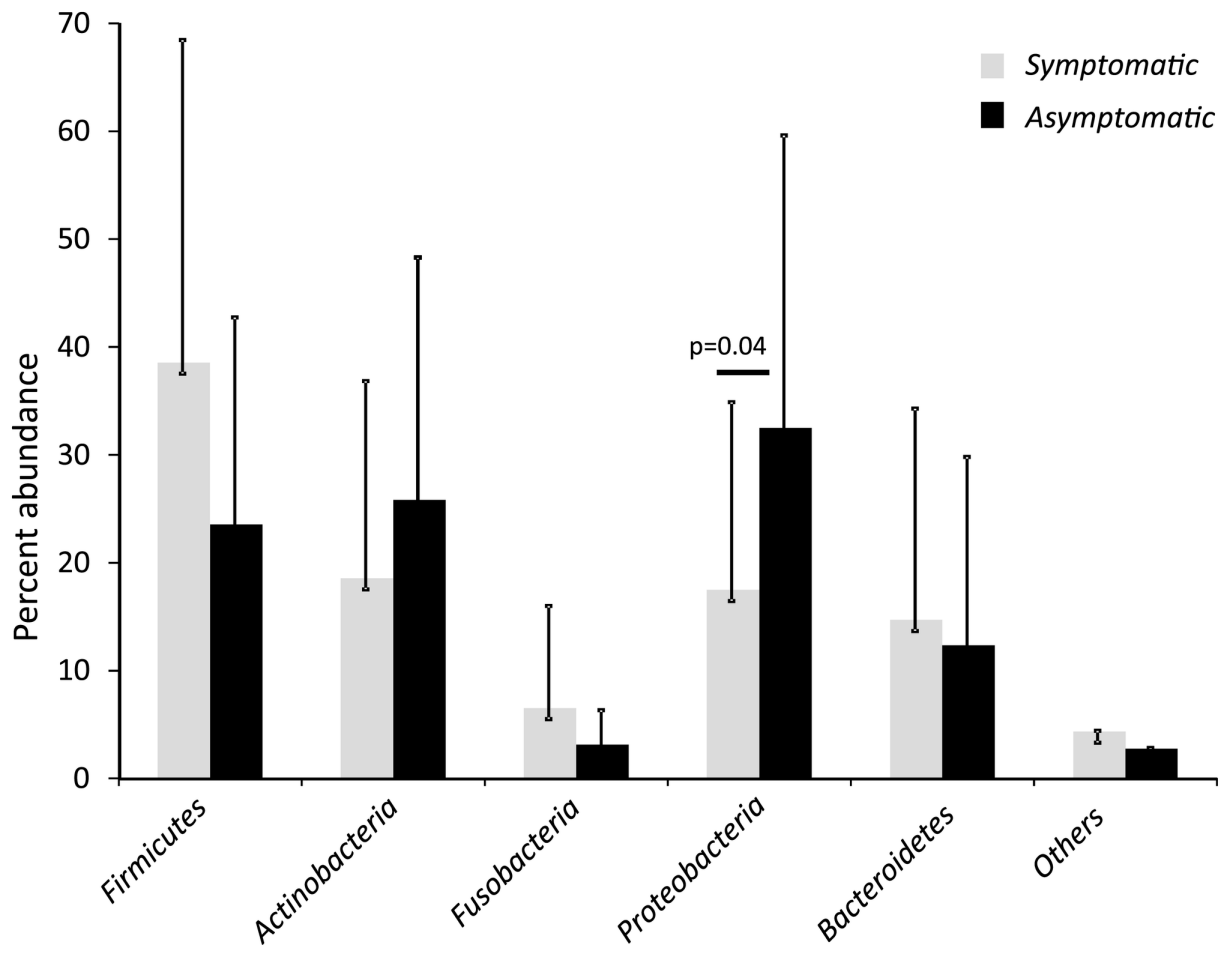
Abundance of the different bacterial phyla in symptomatic (black) and asymptomatic (grey) secondary endodontic infections. The relative abundance of the different phyla according to the disease status is depicted as mean values with standard deviation.

Symptomatic infections had more *Firmicutes* versus asymptomatic infections (38.52% / 23.52%) and more *Fusobacteria* (6.50% / 3.12%) together with less *Proteobacteria* (17.45% / 32.48%) and less *Actinobacteria* (18.53% / 25.81%) than asymptomatic infections. The differences between the two groups were significant for the *Proteobacteria*. Figure 2 shows the relative abundance of the different phyla according to the disease status as mean values with standard deviation. Members of the phyla *Firmicutes*, *Actinobacteria* and *Proteobacteria* were prevalent in all samples, members of *Bacteroidetes* in all but one sample. 

Overall 277 different genera were detected, the most abundant being *Streptococcus* (10.9%), *Prevotella* (8.21%), *Lactobacillus* (8.06%), *Kocuria* (5.17%) and *Neisseria* (3.38%) (see table S1). Some sequences (0.58%) could not be assigned to any taxon. *Enterococcus* was among the 15 most abundant genera (2.59%). 

**Figure 3:**
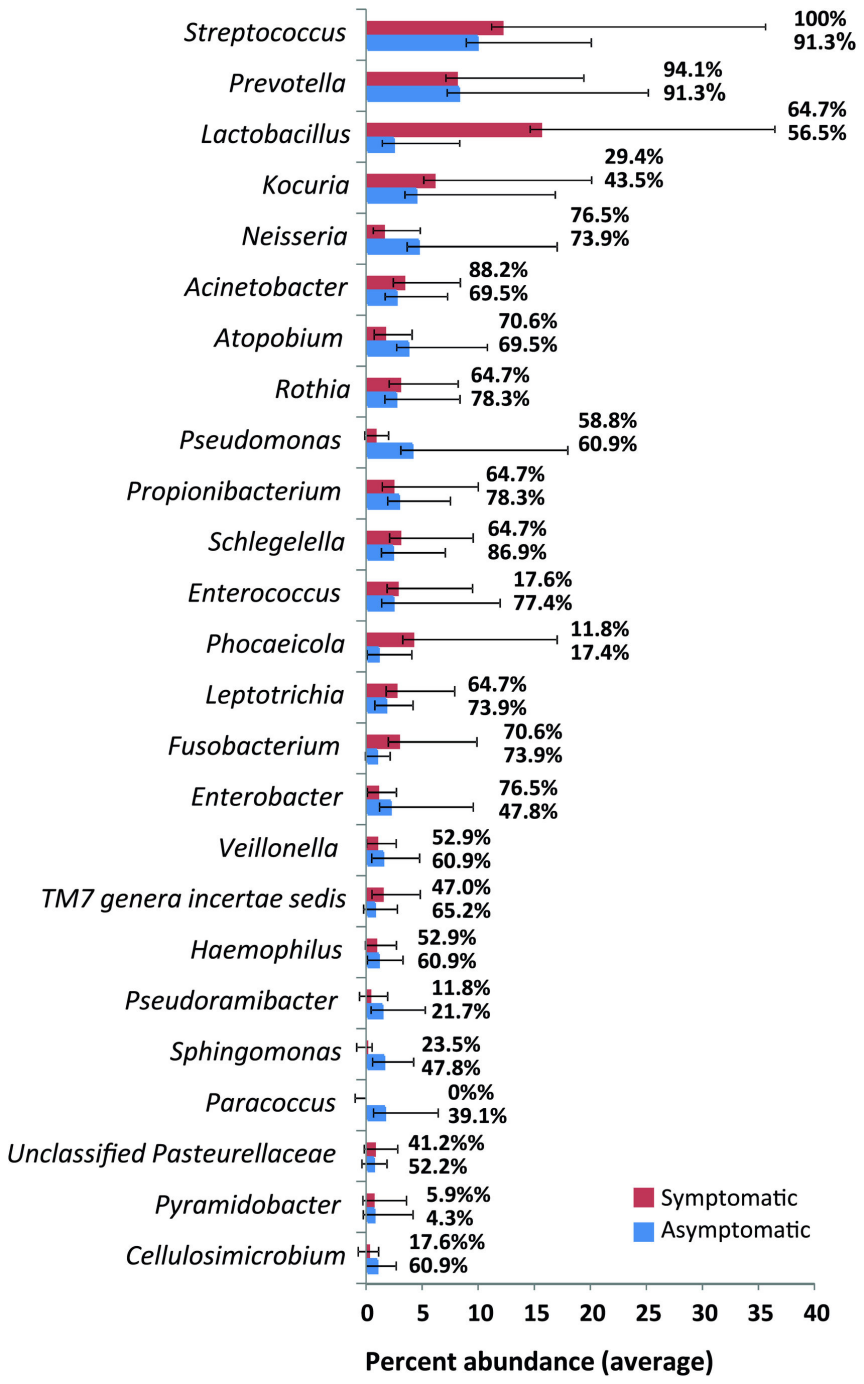
Summarizes the distribution of the top 25 most abundant genera in symptomatic (red) and asymptomatic cases (blue) with their relative abundance data (depicted as bars) and prevalence values (given as numbers next to bars).

**Figure 4:**
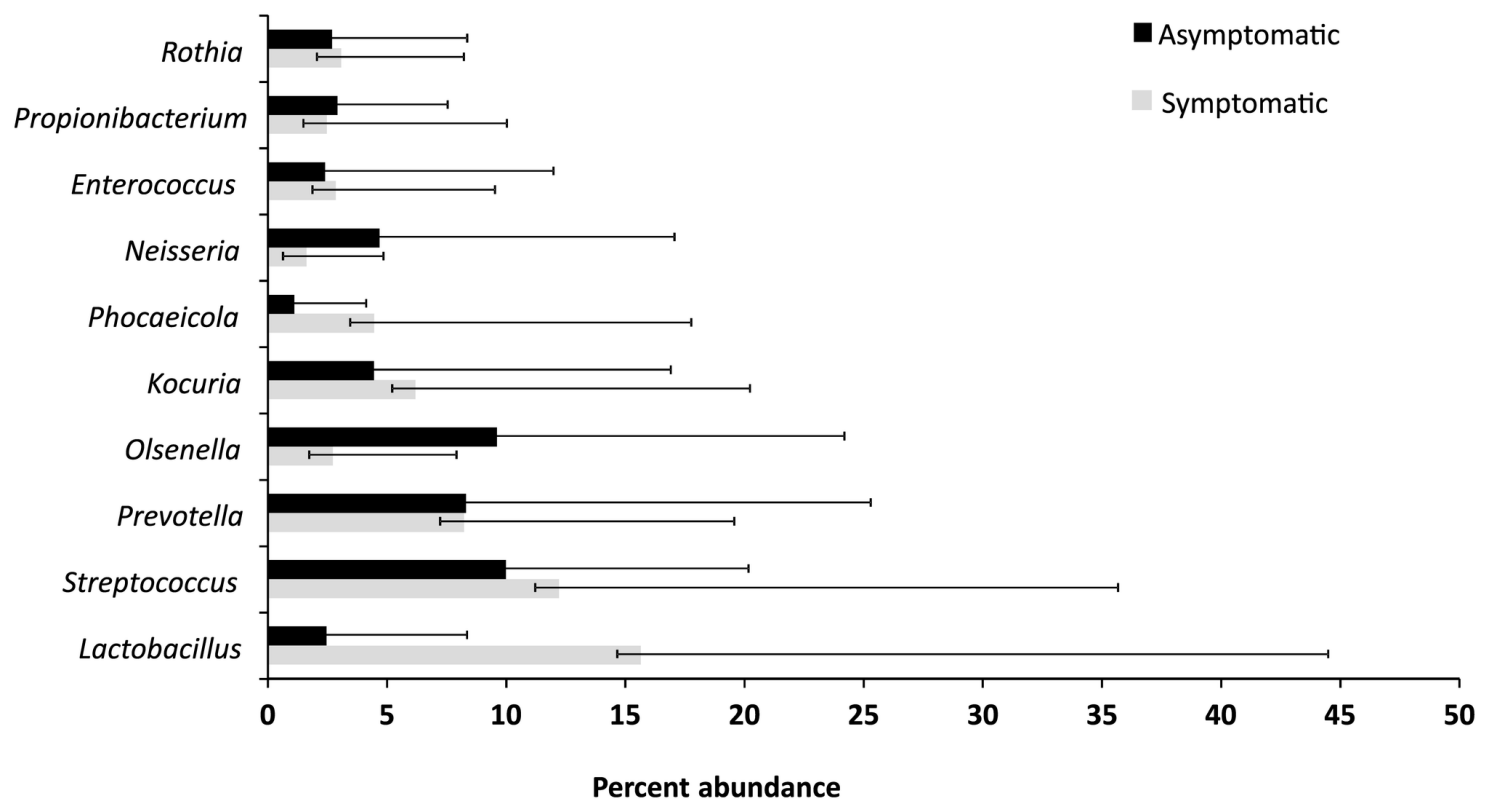
Depicts the mean percentage abundance of the top ten bacterial taxa contributing to more than 52.6% dissimilarity among the two types of samples (symptomatic = black, asymptomatic = grey) from previously root-filled teeth as identified by Bray-Curtis model of SIMPER analysis.

Members of the genus *Streptococcus* were prevalent in all symptomatic cases and in 21/23 asymptomatic cases. Table S1 lists the 25 most abundant genera found in all 40 samples of root canal treated teeth with their abundance and prevalence data. Figure 3 summarizes the distribution of the top 25 most abundant genera in symptomatic and asymptomatic cases with their overall abundance and prevalence values. *Lactobacillus*, *Streptococcus*, *Prevotella*, *Olsenella*, and *Kocuria* were identified to be the top ten bacterial taxa contributing to more than 52.6% dissimilarity among two types of samples (Figure 4).

### OTU analysis of bacterial communities in symptomatic and asymptomatic patients

Overall 18,000 analysed sequences from 40 samples were assigned to 741 species-level OTUs (see Table S2). The symptomatic and asymptomatic cases harboured 413 OTUs and 588 OTUs (3% dissimilarity). Out of 741 OTUs, 260 were shared in both groups while 328 and 153 were uniquely observed in asymptomatic and symptomatic patients (see Figure S1). The mean number of OTUs (3% dissimilarity) was 58 (range 8-104, median 63) for the symptomatic cases and 66 for the asymptomatic cases (range 14-117, median 65). At 5% dissimilarity, a total of 525 OTUs were observed, 216 were shared in both groups, while 213 and 96 were uniquely observed in asymptomatic and symptomatic patients (see Figure S1).

Table 2 lists the diversity and richness estimates. The influence of disease status, age or filling on microbial diversity was analyzed by using the Simpson and Shannon inverse indexes. Both parameters take into consideration the presence and absence of OTUs as well as the abundance of OTUs in the samples. Both calculations showed no significant differences in microbial diversity between asymptomatic and symptomatic sample-associated bacterial communities. 

**Table 2:** Sequencing data and diversity and richness estimate calculations for bacterial taxa in secondary/ persistent endodontic infections with periradicular lesions.

**Indicator**		
Total number of sequences	18,000	
Total OTUs at 3% dissimilarity	741	
Total OTUs at 5% dissimilarity	525	
**3% dissimilarity (95% CI)**	Symptomatic*	Asymptomatic**
Mean number of species-level OTUs per sample (range)	58.53 (8-105)	66.52 (14-117)
Chao1 richness estimator (95% CI)	105.6 (77.11 ; 181.27)	118.87 (88.89; 191.36)
ACE richness estimator (95% CI)	137.6 (107.71; 188.48)	161.09 (126.16; 220.99)
Inverse Simpson index (95% CI)	10.40 (8.96; 12.43)	11.54 (9.92; 13.68)
Nonparametric Shannon estimator	2.77	2.97
Good’s estimator of coverage (%)	94	93
**5% dissimilarity (95% CI)**	Symptomatic*	Asymptomatic**
Mean number of genus-level OTUs per sample (range)	51.47 (8-83)	57.91 (13-102)
Chao1 richness estimator (95% CI)	81.18 (62.29; 135.47)	102.21 (75.12; 174.06)
ACE richness estimator (95% CI)	101.72 (79.63 ; 145.60)	136.59 (105.46; 191.99)
Inverse Simpson index (95% CI)	9.88 (8.57; 11.68)	10.66 (9.21; 12.61)
Nonparametric Shannon estimator	2.66	2.84
Good’s estimator of coverage (%)	95	94
Phylodiversity	6.71	7.18

^*^ values presented are average of 17 patients

^**^ values presented are average of 23 patients

Based on the Chao 1 estimator of richness there are predicted 105.6 at 3% dissimilarity OTUs for the symptomatic and 118.87 for the asymptomatic cases. Calculation of the ACE values estimates 137.6 and 161.09 OTUs at 3% dissimilarity for the symptomatic and asymptomatic cases respectively. The Good’s estimator of coverage revealed 94% and 93% coverage for the data on symptomatic and asymptomatic infections resp. (3% dissimilarity). Rarefaction curves are provided as supporting information (see Figure S2). 

### Bacterial communities and their relation with patient characteristics

**Figure 5:**
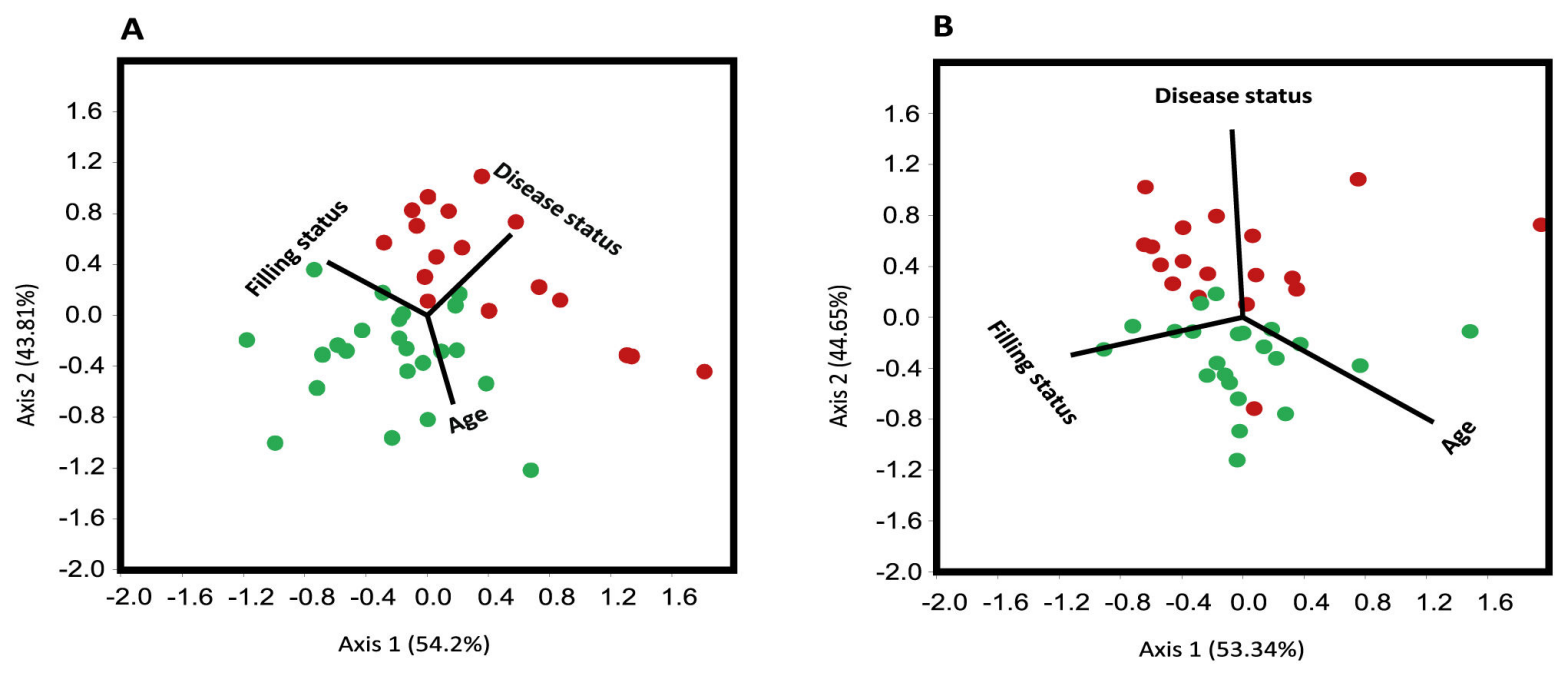
Canonical Correspondence Analysis of symptomatic and asymptomatic secondary root canal infections on species-level (A; 3% dissimilarity) and genus-level (B; 5% dissimilarity). The samples group together according to the disease status (symptomatic – asymptomatic) with the factors ‘age’ and ‘filling status’ taken into account (red = symptomatic, green = asymptomatic).

In order to understand the influence of patients associated environmental variables on microbial communities, Canonical Correspondence Analysis was performed using ‘disease status’ (symptomatic – asymptomatic), age (<30 years and >30 years) and ‘adequacy of filling’ (i.e. if the previous filling exhibited insufficiencies) as a categorical environmental factor. A clear clustering of the samples according to the disease status, i.e. symptomatic and asymptomatic samples was seen even if the analysis was performed on 3% dissimilarity (Figure 5A) or 5%-dissimilarity OTUs (Figure 5B), approximately corresponding to species-level and genus-level respectively. 

In order to see if these factors (‘disease status’, ‘age’ and ‘adequacy of filling’) significantly contributed to the observed differences or not, one-way ANOSIM was performed on 3% dissimilarity and 5%-dissimilarity OTUs observed in symptomatic and asymptomatic patients. Abundance based Bray Curtis (p=0.229, R=0.27) and non-abundance based Jaccard (p=0.02, R=0.021) dissimilarity matrices were applied and showed no significant differences.

**Figure 6:**
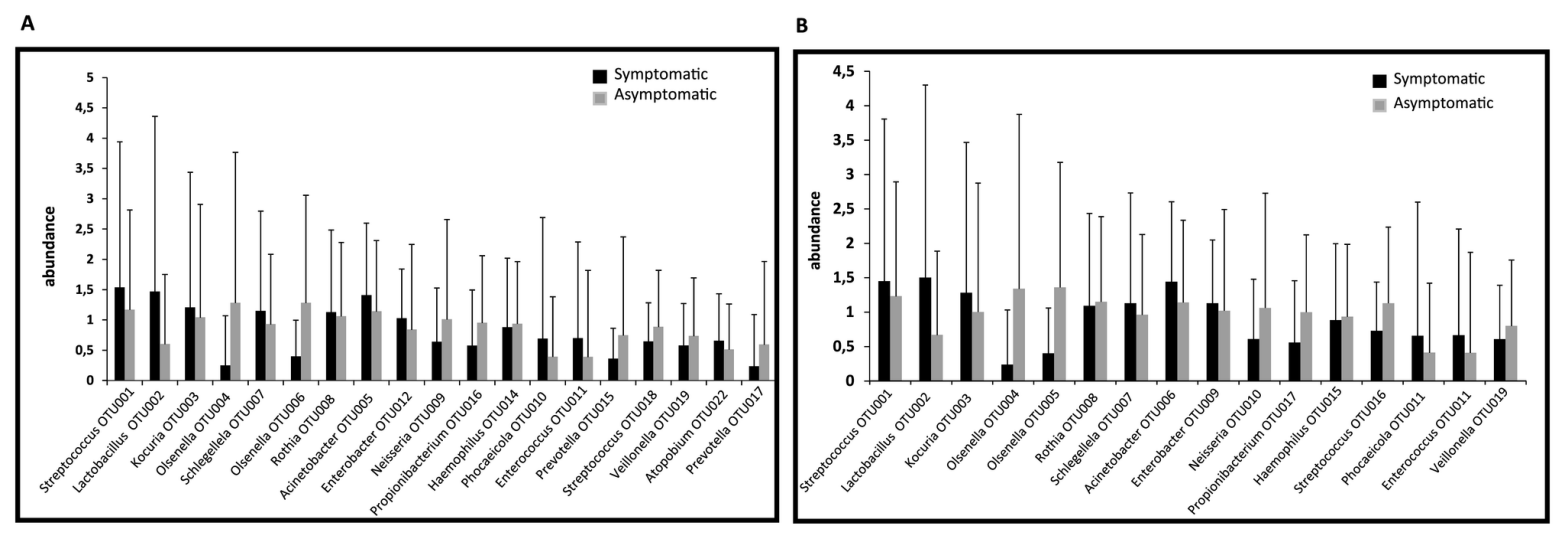
SIMPER Analysis (Bray-Curtis model): Mean percentage abundance (square root transformed) values of species-level OTUs (A; 3% dissimilarity) and genus-level OTUs (B; 5% dissimilarity) contributing to differences in symptomatic versus asymptomatic cases of secondary and persistent root canal infections.

Subsequently two-way ANOSIM was performed for all pairwise combinations of the factors disease status, filling status and age. Two-way ANOSIM applying the Bray-Curtis coefficient (for the 3% dissimilarity data) showed marginal differences (p=0.054, R= 0.11) in bacterial composition of the secondary endodontic infections while using age and disease status as factors. Furthermore SIMPER (similarity percentage) analysis was performed to delineate specific OTUs contributing to the differences between symptomatic and asymptomatic cases. The contribution of the individual OTUs is shown in Figure 6. The following sixteen species level OTUs (3% dissimilarity) accounted for 25% contributions (overall dissimilarity value: 83.82): *Streptococcus* OTU001 (2.35%), *Lactobacillus* OTU002 (2.19%), *Kocuria* OTU003 (1.95%), *Olsenella* OTU004 (1.79%), *Schlegelella* OTU007 (1.47%), *Olsenella* OTU006 (1.23%), *Rothia* OTU008 (1.44%), *Acinetobacter* OTU005 (1.35%), *Enterobacter* OTU012 (1.25%), *Neisseria* OTU009 (1.22%), *Propionibacterium* OTU016 (1.21%), *Haemophilus* OTU014 (1.15%), *Phocaeicola* OTU010 (1.09%), *Enterococcus* OTU011 (1.05%), *Prevotella* OTU015 (0.95%), *Streptococcus* OTU018 (0.94%), *Veillonella* OTU019 (0.92%), *Atopobium* OTU022 (0.84%) and *Prevotella* OTU017 (0.81%) (Figure 6 A). 

On the genus-level (5% dissimilarity) the following OTUs accounted for 25% contributions (overall dissimilarity value: 80.39): *Streptococcus* OTU001 (2.53%), *Lactobacillus* OTU002 (2.43%), *Kocuria* OTU003 (2.14%), *Olsenella* OTU004 (2.02%), *Olsenella* OTU005 (1.68%), *Rothia* OTU008 (1.60%), *Schlegelella* OTU007 (1.58%), *Acinetobacter* OTU006 (1.51%), *Enterobacter* OTU009 (1.48%), *Neisseria* OTU010 (1.37%), *Propionibacterium* OTU017 (1.35%), *Haemophilus* OTU015 (1.26%), *Streptococcus* OTU016 (1.22%), *Phocaeicola* OTU011 (1.17%), *Enterococcus* OTU012 (24.48%) and *Veillonella* OTU019 (25.6%) (Figure 6 B).

## Discussion

This is the first study to compare the bacterial composition of persistent/secondary endodontic infections of a large number of patients with and without symptoms using pyrosequencing. Persistent microorganisms or secondary infections in previously filled root canals correspond to a persistence of periradicular lesions [1,2,27] calling for a revision of the endodontic treatment. The findings of our study revealed alteration of microbial composition and structure in symptomatic and asymptomatic secondary endodontic infection. To date this has not been demonstrated. We demonstrate that secondary infections harbour more diverse microbiota than previously thought. 

Next generation sequencing methods, such as pyrosequencing, are a methodologically sound approach to study human or environment related microbial communities. More recently several studies have been conducted on human associated samples, including the oral cavity, the gut, the skin etc. [31,32]. Microbial community profiling using 16S rRNA gene sequences combined with high-throughput sequencing provides the opportunity to further investigate and analyze the microbiome more accurately, and enables us to study several samples and a broad spectrum of microbial communities simultaneously [1,3,28,29,32]. Yet with the analysis of the sequence data there is a risk of overestimating diversity [46]. Stringent quality control has to be applied to prevent bias [47]. In our study we sequenced 50 samples although the number of sequences in 10 samples was below 450. For deeper microbiota analysis 40 samples with at least 450 sequences were selected to avoid the diversity or composition biases because of the differences of sequence numbers within samples. Only well curated and quality controlled sequences were used for down-stream analysis. 

To date pyrosequencing has only been applied in the study of primary endodontic infections [32,33,36], on small sample sizes and specifically to analyse microorganisms in the apical and coronal part of the root canal (in primary infections) [35,36]. Secondary and persistent endodontic infections have mainly been studied using culture methods [2–4,8,10,13,14,16,17,19] and molecular methods such as species-specific PCR and real-time PCR, checkerboard hybridization and DGGE [5,7,15,20–24]. Only few open-ended 16S rDNA-based cloning studies have been published in recent years [6,25–27]. These have revealed a greater diversity of the microbiota in root-filled teeth with periapical lesions than previously envisaged and a distinctly different bacterial composition compared to primary infections with a higher abundance of gram-positive and facultative anaerobe species [11]. Overall, secondary and persistent endodontic infections were reported to harbour 7 phyla and 58 genera, the main phyla being *Firmicutes*, *Actinobacteria* and *Proteobacteria* [11]. In our study, pyrosequencing of 16S rDNA-sequences from 40 clinical samples allowed a deeper characterization of the bacterial community. The sequences could be assigned to 14 different phyla and 277 genera. The most abundant phyla were *Firmicutes*, *Proteobacteria*, *Actinobacteria*, *Bacteroidetes* and *Fusobacteria* which is in consonance with previous reports [11,18,27]. Six new phyla, not previously identified in root-filled teeth, were found: *TM7*, a candidate division found in environmental samples [48] and recently detected in the oral cavity [49]; *Deinococcus*-*Thermus*, known from extreme environments and recently found in the human stomach [50]; *Cyanobacteria* and *Chloroflexi*, known to live in water and soil; SR1, originally found in marine, terrestrial and freshwater environments and also associated with animals [51] and recently detected in the human oral cavity with an increased abundance in periodontal disease [52] and *OD1*, a newly recognized phylum, only known by its 16S rDNA sequences [53]. These phyla constitute 1.5% of all sequences. 

*Streptococcus*, *Prevotella* and *Lactobacillus* represented the most abundant genera in this study, together constituting more than 25% of all sequences, with *Streptococcus* also representing the most prevalent genus, found in all symptomatic and 21/23 asymptomatic cases. In part this is in accordance with earlier research reporting frequent findings of the genera *Streptococcus*, *Actinomyces*, *Lactobacillus* and *Propionibacterium* [1,27]. The differences might be due to different methodical approaches for sampling and detection, different clinical conditions or geographic differences. In contrast to previous studies discussing *Enterococcus faecalis* as the most prevalent species linked with secondary and persistent endodontic infections [17,19,22], the genus *Enterococcus* was only present in 17.5% of all samples though it was among the 25 most abundant genera. Our results resemble those suggesting a previous overestimation of this opportunistic pathogen as a predominant species [24,54], and rather affirming the role of streptococci as the predominant group. Furthermore, *Enterococcus* was not detected as a single genus in the samples but in association with other taxa, therefore previously reported pure cultures of *Enterococcus faecalis* in endodontic samples [55] could be attributed to the fact that low-abundance members might have escaped cultural detection.

Certain taxa have been associated with the manifestation of characteristic symptoms, i.e. pain, history of pain, tenderness to percussion, particularly in primary but also in secondary endodontic infections [56]. Specifically members of the genera *Prevotella*, *Peptostreptococcus* and *Porphyromonas* were found to be associated with symptoms (in secondary infections) in studies using culture methods and species-specific PCR [14,17,23]. However, the pyrosequencing approach did not provide any results that showed associations of these genera with symptomatic cases. Most likely this might be due to the differences in the methodical approach, and geographic differences might have an additional effect. Yet a noticeably higher abundance of the genus *Lactobacillus* was found in symptomatic samples compared to the asymptomatic samples. Representatives of the genus *Lactobacillus*, also of etiologic importance in forms of dental caries, have been found in many studies analyzing secondary endodontic infections [6,8,16,17,27]. Even though representatives of this genus are generally considered “innocent commensals”, frequent in saliva, they are common in endodontic infections, supporting the assumption made by Siqueira and Rôças, that in this case pathogenicity is dependent rather on the bacterial community in its entirety than the presence of “pathogenic” species per se [57].. Bacterial interactions in the endodontic biofilm can result in additive or synergistic effects among species in mixed communities. It is possible that the *Lactobacilli* might have an effect to promote the growth of other, more virulent species in the community to contribute to the pathogenicity by fulfilling their ecological role. 

Applying the OTU approach the study yielded 741 OTUs at 3% dissimilarity, indicating a substantially higher diversity than the previously reported 158 bacterial species in secondary endodontic infections [11]. The results of the richness estimates point in the same direction, assessing even greater species richness. The symptomatic cases yielded 413 species-level OTUs (3% dissimilarity) whereas the asymptomatic cases yielded 588. The diversity in asymptomatic cases appears slightly greater than in symptomatic cases, although this difference was not statistically significant. This result contradicts the findings of Santos et al [34] who observed a significantly higher diversity in acute primary infections compared to chronic primary infections. 

Pyrosequencing studies comparing health- and disease-associated microbiota in different body sites and infections report very different outcomes concerning bacterial diversity. Analysis of microbiota in bacterial vaginosis and periodontitis revealed a bacterial community with a greater diversity associated with the disease state than with the healthy controls [52,58]. On the other hand, studies of intestinal bacterial communities and microbiota in severe caries report contrary findings: Carroll I (2012 [59]) found significantly decreased bacterial diversity in samples from patients with irritable bowel syndrome compared with healthy controls. Similarly Manichanh et al. found decreased bacterial diversity in patients with Crohn’s disease [60]). A study of plaque from patients with severe caries revealed a decrease in bacterial diversity as caries progressed from healthy to cavitated and to deep dentinal lesions [61], attributed to the substrate-driven change of pH among other factors.

Different underlying reasons such as environmental factors or bacterial synergisms and antagonisms and the “original” microbiota present at the studied site seem to contribute to these contrasting findings. In our study we compared symptomatic versus non-symptomatic infections, i.e. different disease states, where the symptomatic state is more progressed. There might also be a change in environmental variables (pH, substrate availability) in the root canal during progression of the infection responsible for the slight decrease in diversity.

Comparing symptomatic and asymptomatic cases with multivariate analysis also revealed differences in the composition and prevalence of different members of the microbial community dependent on the disease status. A Canonical Correspondence Analysis, considering the factors age, filling status and disease status shows a grouping of samples according to the disease status. Similarly the two-way ANOSIM revealed some differences in bacterial composition of the root canal samples from symptomatic and asymptomatic infections in association with patient age at the time of sampling.

 Most research that has associated the composition of human microbiota with the factor age has been done on the intestinal microbiota. Britton et al 2013 conclude that there is conflicting data on the changes of the gut microbiome with ageing [62]. Some studies report no significant changes between young (30 yr) and older people (70 yr) [63]. However several other studies report a strong influence of the factor age on the composition of the bacterial community [64-66]. To date there are but a few in depth studies investigating the influence of age on the oral microbiome, mostly during childhood. Crieelard et al analyzed the salivary microbiota of children 3-18 years old. They found differences in the proportions of several phyla in children compared to adults as well as an increase of members of the genus *Veillonella*, *Selenomonas* and *Prevotella* with age and conclude a maturation of the oral microbiome from the early childhood through puberty until adulthood [67]. Stahringer et al studied saliva samples from twins and confirmed the changes of the salivary microbiota over time from the age of 8-26. They reported greater changes in puberty (12-17yrs) than during adolescence (17-22) and also found age-specific abundance profiles for members of the genera *Veillonella*, *Actinomyces* and *Streptococcus* [68]. 

The age range of the patients analyzed in this study was from 18-56 yr, so all of the samples would represent an “adult” microbial flora. Nevertheless the factor age associated with the factor disease status influenced the composition of the bacterial community found in the root canal samples. Since the samples were obtained from patients from the Sudan, the circumstance of the patients coming from a very poor developing country has to be considered. Here differences in the health care system, hygiene standard and oral health might have an effect on the oral microbiome dependent on the age.

Yet more importantly the samples in this study were taken from endodontic infections with the pulp space originally representing a microbe-free space. That means there is no “healthy microflora” as in saliva, even though the bacterial species that find their way down into the root canal might derive from the salivary microbiota. For this reason it can only be assumed that patients of different age groups could have different endodontic microbiota due to differences in their salivary microbiota.

The RDP classification of our sequence data detected substantially more members of the phyla *Firmicutes* and *Fusobacteria* in symptomatic cases whereas asymptomatic patients harboured significantly more *Proteobacteria* and more *Actinobacteria*. These results point at the bacterial community and its composition as the unit responsible for the manifestation and characteristics of the secondary endodontic infections rather than one or few pathogenic species. A number of studies have dealt with the concept of “bacterial community-based pathogenicity” in different diseases, particularly oral infections [57,69]. It is assumed that rather than being caused by one pathogenic microorganism, they are attributed to the interactions within a specific bacterial community or the fact that specific niches in an environment are occupied. That way the community as a collective plays a role in the pathogenesis [70]. Not only highly abundant but also low abundant species can act a relevant part in this scenario. Only recently next generation sequencing techniques made it feasible to capture the low abundant members of bacterial communities in the investigated environments. This study disclosed a great amount of low abundant OTUs in secondary endodontic infections. Also several new phyla were detected that were present in low abundance. These low-abundant members e.g. *TM7* may be responsible for differences in the composition of the bacterial community and provide specific niches for other species that may lead to a higher virulence of the whole community [33,71]. In light of the data analysed, secondary endodontic infections could be ranked among the endogenous infections that are characterised by a polymicrobial etiology with the bacterial community, mostly organised in a biofilm, as the chief culprit.

## Conclusion

Using the high-throughput pyrosequencing approach, an overall greater diversity of bacterial taxa in secondary endodontic infections was observed than previously reported, including many low-abundant members and several phyla reported for the first time. Our results showed that the composition of the bacterial community was altered in patients with and without symptoms, a trend of reduced diversity was observed, although these differences were not statistically significant.

Considering the influence of the bacterial community as a whole on the incidence of disease, treatment protocols could certainly be adapted and improved. Treatment strategies should still aim at a most thorough elimination of the microbiota residing in the infected root canal, with a focus on disturbing the ecosystem to prevent the survival of the bacterial community. Additionally, alternative measures to eradicate resistant microorganisms such as passive ultrasonic irrigation, apical negative pressure irrigation systems, ozone gas and photodynamic therapy may provide helpful treatment options in addition to chemo-mechanical preparation [72–75].

## Supporting Information

Table S1 **Table showing the 25 most abundant genera found in 40 samples of root canal treated teeth with their overall abundance (standard deviation in brackets) and prevalence data.**
(DOCX)

Table S2 **Excel file with OTUs at 3% dissimilarity, A) and OTUs at 5% dissimilarity; B) found in 40 samples of root canal treated teeth.**
(XLSX)

Figure S1 **Diagram showing shared OTUs of symptomatic and asymptomatic secondary root canal infections at 3% dissimilarity (left) and 5% dissimilarity (right).**
(TIF)

Figure S2 **Diagram of rarefaction analysis of V1-V2 pyrosequencing reads of the 16S rRNA gene in samples from 40 previously filled root canals of symptomatic and asymptomatic teeth.** Rarefaction values were generated at a 97% sequence similarity cut-off value by MOTHUR.
(TIF)
