# Supplementary material for: Comparison of the Bacterial Composition and Structure in Symptomatic and Asymptomatic Endodontic Infections Associated with Root-Filled Teeth Using Pyrosequencing
Source: PLoS One. 2013 Dec 30;8(12):e84960. doi: 10.1371/journal.pone.0084960 (PMC3875544; doi:10.1371/journal.pone.0084960)
Supplement: Table S1 — Table showing the 25 most abundant genera found in 40 samples of root canal treated teeth with their overall abundance (standard deviation in brackets) and prevalence data. (DOCX) [file pone.0084960.s001.docx]

**Supporting material Table S1**

The 25 most abundant genera found in 40 samples of root canal treated teeth with their abundance and prevalence data

| **Phylum** | **Genus** | **Abundance [%]** | **prevalence** |
| --- | --- | --- | --- |
| *Firmicutes* | *Streptococcus* | 10.9 (± 0.42) | 38/40 |
| *Bacteroidetes* | *Prevotella* | 8.2 (± 0.37) | 37/40 |
| *Firmicutes* | *Lactobacillus* | 8.06 (± 0.50) | 24/40 |
| *Actinobacteria* | *Kocuria* | 5.17 (± 0.32) | 15/40 |
| *Proteobacteria* | *Neisseria* | 3.38 (± 0.24) | 30/40 |
| *Proteobacteria* | *Acinetobacter* | 3.01 (± 0.12) | 31/40 |
| *Proteobacteria* | *Atopobium* | 2.88 (± 0.14) | 28/40 |
| *Proteobacteria* | *Rothia* | 2.84 (± 0.14) | 28/40 |
| *Proteobacteria* | *Pseudomonas* | 2.73 (± 0.26) | 24/40 |
| *Actinobacteria* | *Propionibacterium* | 2.72 (± 0.15) | 26/40 |
| *Actinobacteria* | *Schlegelella* | 2.67 (± 0.14) | 31/40 |
| *Firmicutes* | *Enterococcus* | 2.59 (± 0.21) | 7/40 |
| *Bacteroidetes* | *Phocaeicola* | 2.43 (± 0.22) | 6/40 |
| *Fusobacteria* | *Leptotrichia* | 2.19 (± 0.10) | 28/40 |
| *Fusobacteria* | *Fusobacterium* | 1.81 (± 0.12) | 29/40 |
| *Proteobacteria* | *Enterobacter* | 1.72 (± 0.14) | 24/40 |
| *Fimicutes* | *Veillonella* | 1.29 (± 0.07) | 22/40 |
| *TM7* | *TM7_genera incertae sedis* | 1.08 (± 0.07) | 23/40 |
| *Proteobacteria* | *Hämophilus* | 1.03 (± 0.05) | 23/40 |
| *Firmicutes* | *Pseudoramibacter* | 1.0 (± 0.08) | 7/40 |
| *Proteobacteria* | *Sphingomonas* | 0.97 (± 0.05) | 16/40 |
| *Proteobacteria* | *Paracoccus* | 0.96 (± 0.09) | 9/40 |
| *Proteobacteria* | *Unclassified Pasteurellaceae* | 0.73 (± 0.04) | 19/40 |
| *Synergistes* | *Pyramidobacter* | 0.72 (± 0.08) | 2/40 |
| *Actinobacteria* | *Cellulosimicrobium* | 0.69 (± 0.04) | 17/40 |
