# Supplementary figures and images for: Comparison of the Bacterial Composition and Structure in Symptomatic and Asymptomatic Endodontic Infections Associated with Root-Filled Teeth Using Pyrosequencing
Source: PLoS One. 2013 Dec 30;8(12):e84960. doi: 10.1371/journal.pone.0084960 (PMC3875544; doi:10.1371/journal.pone.0084960)

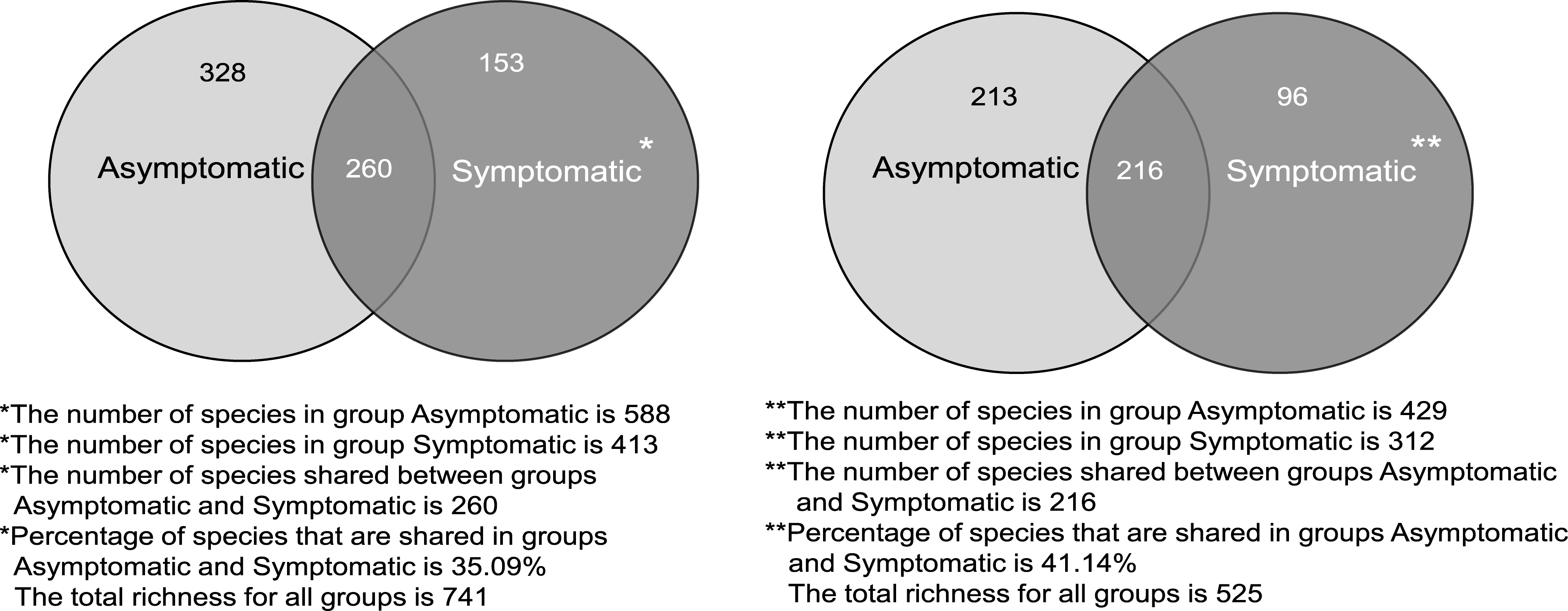

Supplement: Figure S1 — Diagram showing shared OTUs of symptomatic and asymptomatic secondary root canal infections at 3% dissimilarity (left) and 5% dissimilarity (right). (TIF) [file pone.0084960.s003.tif]

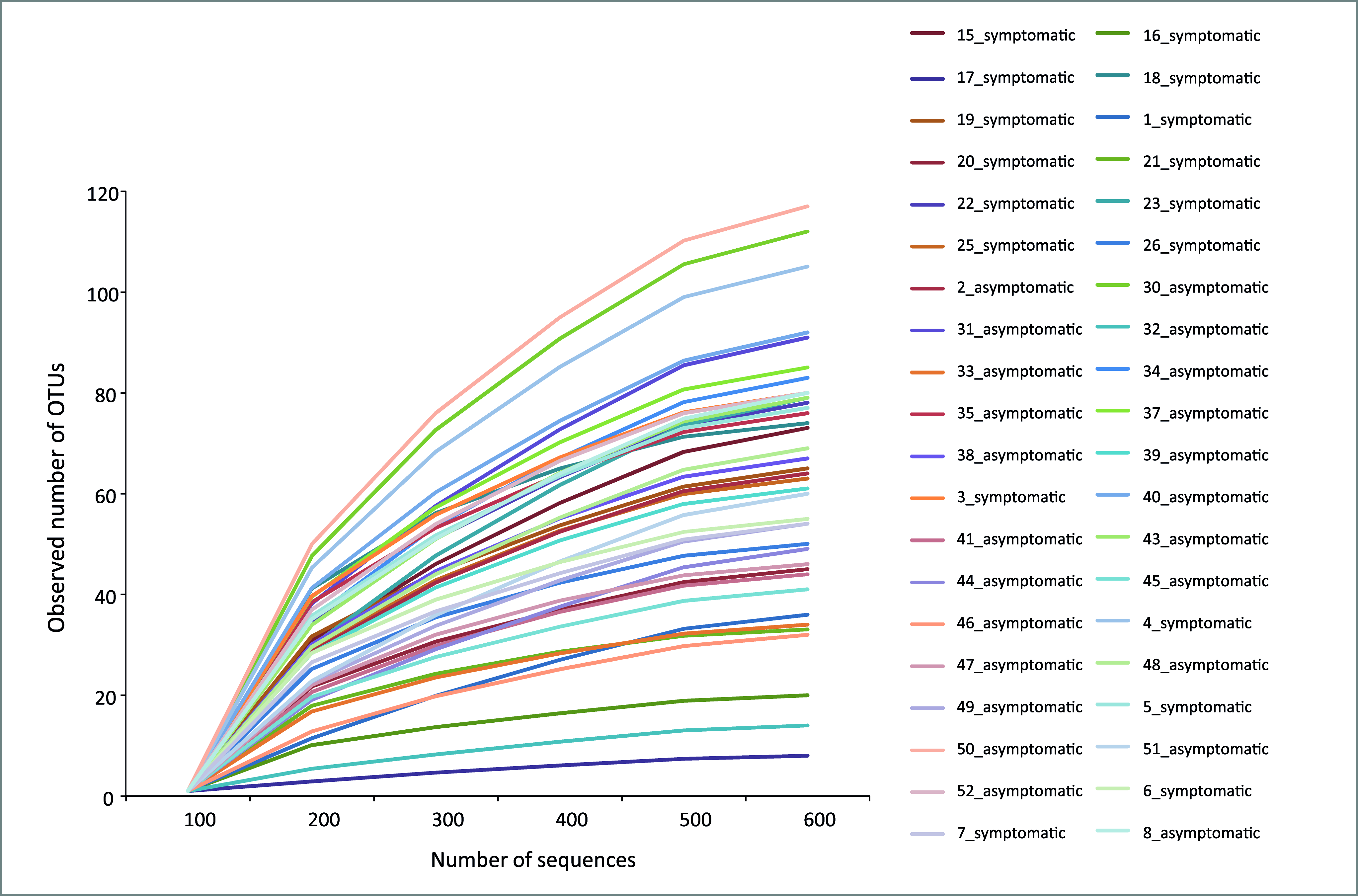

Supplement: Figure S2 — Diagram of rarefaction analysis of V1-V2 pyrosequencing reads of the 16S rRNA gene in samples from 40 previously filled root canals of symptomatic and asymptomatic teeth. Rarefaction values were generated at a 97% sequence similarity cut-off value by MOTHUR. (TIF) [file pone.0084960.s004.tif]
